# Production of bikaverin by a *Fusarium fujikuroi* mutant in submerged cultures

**DOI:** 10.1186/s13568-016-0205-0

**Published:** 2016-05-03

**Authors:** G. J. Lale, R. V. Gadre

**Affiliations:** Biochemical and Biological Engineering Group, Chemical Engineering and Process Development Division, CSIR-National Chemical Laboratory, Pune, 411008 India

**Keywords:** Bikaverin, *Gibberella fujikuroi*, *Fusarium fujikuroi*, Submerged cultures, Pigment production

## Abstract

A variety of mutants having different colony characteristics, morphology and soluble pigmentation were generated from *Fusarium fujikuroi* by exposure to UV radiation. Mutants were selected that formed dry, compact, small colonies with reddish-violet pigment on regeneration agar plates. The production of bikaverin by Mut-4 was examined in shake flasks in media with different nitrogen and carbon sources. The optimal C: N ratio for the maximal bikaverin production by Mut-4 was 150:1. It produced still higher bikaverin (6.3 g l^−1^) in a medium containing defatted cottonseed meal as nitrogen source, in combination with glucose. Bikaverin produced was extracted, purified and characterized by UV-visible and NMR spectroscopy. Bikaverin production in the present investigation was substantially higher than that reported by earlier investigators in submerged and solid-state fermentations.

**Electronic supplementary material:**

The online version of this article (doi:10.1186/s13568-016-0205-0) contains supplementary material, which is available to authorized users.

## Introduction

Production of pigments by fungi is an interesting phenomenon because of their wide variations and potential applications as reviewed (Mapari et al. 2010; Celestino et al. 2014; Akilandeswari and Pradeep 2016). The majority of the fungal pigments produced belong to the group of compounds like quinones, flavonoids, melanins and azaphilones, which are aromatic polyketides and are reported to have medicinal uses as well as potential dyes (Kongruang 2011). Bikaverin is a reddish polyketide pigment produced by *Gibberella fujikuroi* in addition to large amounts of gibberellins (Limon et al. 2010). *G. fujikuroi* is now renamed as *Fusarium fujikuroi*. The fungus produces a variety of secondary metabolites like moniliformin, beauvericin, fumonisin, fusarin C neurosporaxanthin and the red pigment bikaverin as well as its isomer, nor-bikaverin (Fotso et al. 2002). Although the functions of most secondary metabolites are unknown, it is generally recognized that pigments are likely to protect fungi from exposure to environmental stresses such as irradiation and oxidation which result in their growth inhibition while other compounds contribute to the fungal virulence (Medentsev et al. 2005).

Many filamentous fungi produce polyketide-derived pigments, which have been studied for their biological activity and taxonomic value. Polyketides are a group of secondary metabolites, exhibiting diversity in terms of their structure and function. These compounds are of immense interest due to their pharmaceutical importance. Their wide-spectrum biological activities make them economically, clinically and industrially useful compounds (Hertweck 2009).

Bikaverin, the bright red pigment, is a polyketide in nature (Kjaer et al. 1971; Linnemannstöns et al. 2002). It has also been found to affect different biological processes. It exhibits anti-protozoal activity against *Leishmania brasilensis* at 0.15 µg per ml concentration (Balan et al. 1970). This inhibitory activity is also observed against mammalian cell lines and it has been reported that bikaverin is cytotoxic to Ehrlich ascites carcinoma, lymphoma L5178Y, sarcoma 37, lymphoadenoma NK/LY, sentinel pancreatic cancer cell line MIA Pa Ca-2 and HeLa cells (Fuska et al. 1975). These effects are presumably due to inhibition of ATP synthesis caused by uncoupling of oxidative phosphorylation (Henderson et al. 1977). It also inhibits succinate and NAD-linked respiration in rat mitochondria and causes swelling of the mitochondria (Kitagawa et al. 1997). Bikaverin is also reported to have anti-fungal activity. Aqueous solutions prepared from crystalline bikaverin caused vacuolization in hyphal tips of *Aspergillus niger* and thirty other fungal species (Cornforth et al. 1971). Bikaverin can be used as a biocontrol agent against tomato late blight caused by *Phytophthora infestans* (Kim et al. 2007). It acts as anti-oomycete substance against *P. infestans* (Son et al. 2008). It also shows nematicidal activity against pine wood nematode *Bursaphelenchus xylophilus* causing pine wilt disease in a number of *Pinus* species. At 100 μg l^−1^ concentration, it could kill *B. xylophilus* with 50 % mortality. When applied with fusaric acid at a ratio of 1:1, the mixture had more potent activity and acted synergistically on *B. xylophilus* (Kwon et al. 2007). It is suggested that it could have a role in the pathogenesis of *Fusarium oxyporum* (Bell et al. 2003).

**Fig. 1:**
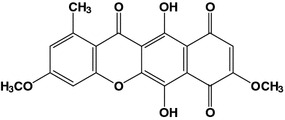
Chemical structure of bikaverin as described by Cornforth et al. (1971)

The structure of bikaverin (6, 11-dihydroxy-3, 8-dimethoxy-1-methyl-benzo-xanthein-7, 10, 12-trion) as shown in Fig. 1 was determined by x-ray crystallography and chemical synthesis (Cornforth et al. 1971). Its chemical synthesis was achieved by condensation of nine acetate units into a single polyketide chain, which was then folded and cross-linked to form the bikaverin (Kjaer et al. 1971).

Bikaverin is produced by *F. fujikuroi* via a polyketide route (Giordano et al. 1999). The cultures acquire a reddish pigmentation because of internal accumulation and excretion of bikaverin into the medium. The production of bikaverin and other secondary metabolites in *F. fujikuroi* is strongly influenced by aeration. Higher aeration is reported to enhance fungal growth as well as the production of bikaverin and gibberellins (Giordano et al. 1999). Production of bikaverin also depends on the type and availability of nitrogen source, carbon source and the pH of the medium as described by Bell et al. (2003). The polyketide synthase gene *bik1* is responsible for synthesis of bikaverin in *F. fujikuroi* (Linnemannstöns et al. 2002). Genetic study revealed that a cluster formed by six *bik* genes encode a multifunctional polyketide synthase enzyme which is involved in the biosynthesis of bikaverin (Wiemann et al. 2009). Recently Wiemann et al. (2013) have presented genome sequence of *F. fujikuroi* and related members of genus *Fusarium* and the details of the genes involved in production of secondary metabolites using bioinformatics and experimental gene sequencing. Their work shows that the *BIK* gene cluster is located on Chromosome-V of *F. fujikuroi*. They are repressed at alkaline pH condition (Linnemannstöns et al. 2002) and by high amount of nitrogen in an AreA-independant manner and are regulated by complex regulatory network (Wiemann et al. 2009).

Though considerable work has been done on bikaverin isolation, confirmation of structure, chemical reactions and biological applications, very few reports are available regarding its production in large enough quantity. Kajer et al. (1971) and Bu’Lock et al. (1974) had studied its production in shake flask cultures by *F. fujikuroi* ACC-917 and *F. fujikuroi* ATCC 12616, respectively. Kumar and Losane (1988) reported production of 25 mg l^−1^ bikaverin by immobilized mycelia of *F. fujikuroi* P-3. It is reported that the shake flask cultures of *F. fujikuroi* U strain produced 458 mg l^−1^ (Balan et al. 1970) while *F. fujikuroi* (Sawada) strain CDBB H-984 produced 3 g l^−1^ bikaverin (Escamilla-Silva et al. 2001). In solid-state fermentation, 187 mg kg^−1^ and 2 g kg^−1^ bikaverin production was reported by cultivating *Fusarium oxysporum* f sp *vasinfectum* (Bell et al. 2003) and *Fusarium verticillioides* KF 419 (Chelkowski et al. 1992), respectively.

Aim of the present study was to investigate bikaverin production by Mut-4, a mutant of *F. fujikuroi* NCIM 1019, cultivated using different carbon, nitrogen sources and the role of C:N ratio on its production in shake flask cultures.

## Materials and methods

### Microorganism

*Fusarium fujikuroi* (NCIM 1019) was obtained from National Collection of Industrial Microorganisms (NCIM), National Chemical Laboratory, Pune 411008, India.

### Chemicals and media ingredients

The media ingredients were from HiMedia (Mumbai, India). Defatted soy bean meal and cottonseed meal were from Chandrasekhar Exports Pvt. Ltd. (Kolhapur, India). Chloroform, methanol, formic acid (85 %) and HPLC grade acetonitrile, ammonium di-hydrogen phosphate and phosphoric acid (AR grade) were from E. Merck (Mumbai, India). Gibberellic acid (G 7645) used as a reference compound for high performance liquid chromatography (HPLC) was purchased from Sigma Chemical Company, St. Louis, MO, USA. Whatman filter papers were purchased from GE Life Science, Mumbai, India. Plastic were used were purchased from Tarsons, Kolkata, India.

### Media and culture conditions

The parent strain and mutants were maintained on potato dextrose agar (HiMedia Mumbai, India) supplemented with 2 g l^−l^ yeast extract. Slants were inoculated and incubated at 28 °C for 4–5 days, and then stored at 4 °C. The regeneration agar for the growth of survivors after mutagenesis contained (g l^−1^) KH_2_PO_4_ 1.5, NaCl 0.5, MgSO_4_·7H_2_O 0.2; Na_2_MoO_4_·2H_2_O 0.05, yeast extract 3.0, glucose 20, soy peptone 3.0, bile salt 1.0, trace mineral solution 1 ml and agar 20.0. The pH was adjusted to 6.8. The trace mineral solution contained (mg l^−1^) H_3_BO_3_ 100, MnCl_2_·4H_2_O 100, ZnSO_4_·7H_2_O 100, FeCl_3_·6H_2_O 100, CaCl_2_·2H_2_O 1000, and CuCl_2_·2H_2_O 50. A few drops of conc. HCl were added to the solution till it became clear.

The cultures were grown in for 5 days at 28 °C with agitation at 200 rpm in 250 ml Erlenmeyer flasks, each containing 20 ml basal fermentation medium with following composition (g l^−1^) KH_2_PO_4_ 1.5, NaCl 0.5, MgSO_4_·7H_2_O 0.2, Na_2_MoO_4_·2H_2_O 0.05, defatted soy bean meal 2, sucrose 20 and trace mineral solution 1 ml. On the third day of incubation, separately autoclaved 500 g l^−1^ sucrose solution was added to the growing fungal cultures so that the total sucrose fed to the shake flask was 50 g l^−1.^ Other carbon sources and nitrogen sources were used on equal “C” and equal “N” basis, respectively after determination of Kjeldahl nitrogen content in organic nitrogen sources. Other details varied with experimental run, as detailed below. All the experiments were done in triplicate and average values were recorded.

### Mutagenesis

The parent strain *F. fujikuroi* NCIM 1019 was grown in 25 ml basal fermentation medium with (g l^−l^) soy bean meal 2 and sucrose 20, for 48 h, at 28 °C, 200 rpm. The mycelium was filtered repeatedly through sterile sintered glass funnels with 50–100 micron pore size, (Borosil, Mumbai, India) to get a cell suspension that contained mycelia fragments with only one or two cells. The short mycelial fragments in the filtrate were counted using a haemocytometer and 20 μl of this suspension was spread on regeneration agar plates to have around 2000 colonies per plate. The plates were exposed to germicidal UV radiation using a UV tube light (Sankyo Denki Co. Ltd., Japan) at 10 cm height, for 3–12 s and incubated in dark, at 28 °C, for 5–7 days. A specially fabricated UV exposure chamber that did not require switching the lamp on and off repeatedly was used for exposing the plates to UV for accurate time interval. The colonies with enhanced pigmentation and different growth characteristics were selected. The selected colonies were maintained on potato dextrose agar slants.

### Production of bikaverin by selected mutants

Tubes containing 2.5 ml basal fermentation medium were inoculated from fresh slants of the parent culture *F. fujikuroi* NCIM 1019 and the selected mutants, generated during mutagenesis. The tubes were incubated at 28 °C, for 48 h, under shaking. Flasks containing basal fermentation medium were inoculated from the tube cultures. The flasks were incubated and the fermentation broth was analyzed for dry cell mass, bikaverin, gibberellic acid and pH.

### Enhancement of bikaverin production in shake flask cultures

In an effort to increase the cell mass concentration and thereby production of bikaverin by the parent *F. fujikuroi* NCIM 1019 and the selected mutant, Mut-4, they were studied using different concentrations of defatted soy bean meal as nitrogen source in the fermentation medium. Defatted soy bean meal was varied from 2 to 10 g l^– l^ in different flasks. The parent strain *F. fujikuroi* NCIM 1019 and Mut-4 were inoculated from fresh slants in 2.5 ml basal fermentation medium tubes and incubated for 48 h at 28 °C, 200 rpm. After 48 h, liquid cultures were transferred to basal fermentation medium in 250 ml Erlenmeyer flask. The flasks were incubated for 48 h and used as inoculum. Flasks containing 20 g l^−l^ sucrose and 2 to 10 g l^−1^ defatted soy bean meal were inoculated with 2.5 ml liquid culture from the respective inoculum flasks. The flasks were incubated and analyzed as mentioned in the earlier experiment. The experiments were performed in triplicate.

### Suitable carbon sources for bikaverin production by mutant Mut-4

Fermentation media containing defatted soy bean meal 2 g l^−1^ (0.13 g l^−1^ N) and different “C” sources at 50 g l^−l^ concentration (20 g l^−1^ “C”) were used. Cellobiose, fructose, galactose, glucose, lactose, maltose, sucrose, xylose and soluble starch were used as carbon sources and added after separate autoclaving. The inocula of selected mutant “Mut-4” were grown in 2.5 ml basal fermentation medium with respective sugars for 48 h, at 28 °C, 200 rpm. The flasks were incubated and at the end of incubation, samples were analyzed for dry cell mass, bikaverin and gibberellic acid.

### Effect of different nitrogen sources on bikaverin production by mutant Mut-4

The fermentation media with different inorganic and organic nitrogen sources were used in the study. Nitrogen sources were examined on equal nitrogen basis (equivalent to 0.13 g l^−1^ “N”). Inorganic nitrogen sources like ammonium nitrate, ammonium sulphate, ammonium chloride and organic nitrogen sources viz. meat peptone, soy peptone, yeast extract, defatted cottonseed meal and soy bean meal were used. Separately autoclaved glucose was added equivalent to 20 g l^−l^ “C” source. The flasks were inoculated with liquid culture of Mut-4 grown in 2.5 ml basal fermentation medium with respective nitrogen sources for 48 h at 28 °C, 200 rpm. The flasks were incubated and analyzed as described earlier.

### Bikaverin production by mutant Mut-4 in cotton seed meal medium

Based on the earlier experiments, a modified fermentation medium composed of (g l^−1^) KH_2_PO_4_ 1.5, NaCl 0.5, MgSO_4_.7H_2_O 0.2, Na_2_MoO_4_.2H_2_O 0.05, with defatted cottonseed meal 2 and glucose 50 was used for bikaverin production by mutant Mut-4. Eighteen Erlenmeyer flasks each containing 22.5 ml modified fermentation medium were inoculated with 2.5 ml, 48 h old liquid cultures of mutant Mut-4. Flasks were incubated and at every 24 h, three flasks were withdrawn for determination of bikaverin, gibberellic acid, pH and dry cell mass. Along with the extracellular bikaverin from the culture filtrate, bikaverin accumulated in the biomass was extracted as follows. The cell mass obtained at every 24 h from the shake flask cultures was lyophilised and bikaverin from this dry mass was extracted by refluxing with chloroform acidified with concentrated HCl, for 6 h. The biomass was separated by filtration and the chloroform layer of the extract was separated. The chloroform was removed under vacuum and bikaverin was analyzed as described below. The mutant is deposited in National Collection of Industrial Microorganisms (NCIM), National Chemical Laboratory, Pune, India as NCIM 1344.

### Analytical methods

At the end of incubation, the volume of the broth was adjusted to 25 ml with distilled water and filtered under vacuum through Whatman filter paper Grade-1. The cell mass was washed with distilled water and dry cell weight was estimated at 103 °C. The filtrates were analyzed for bikaverin, gibberellic acid, pH and residual glucose.

Measured volumes of the culture filtrate obtained from 5-day old cultures were acidified to pH 2.5 with 1 N HCl and partitioned with an equal volume of chloroform repeatedly, until the red pigment was removed from aqueous phase. The chloroform fractions were pooled, chloroform was removed under vacuum and the residue was re-dissolved in an appropriate volume of chloroform. These solutions were filtered through 0.2 μm membrane filters and their absorbance was measured spectroscopically (Spectrascan UV 2600, Chemito, Mumbai, India) and bikaverin was quantified from the peak absorption log €: 3.95, at 518 nm as described by Giordano et al. (1999). The chloroform extract was then reduced in volume and an equal volume of diethyl ether was added, to get a red precipitate. The precipitate was taken in appropriate volume of chloroform and used for thin layer chromatography (TLC) using aluminium sheets coated with silica gel 60 (Merck Life Science, Mumbai, India) and a mobile phase composed of chloroform:methanol:formic acid (85:15:1, v/v) as described by Bell et al. (2003). The coloured bands were eluted from the silica gel with a mixture of chloroform: methanol in the ratio 1:2 (v/v). The resulting solution was filtered, concentrated and pigment identity was confirmed by spectral scan using a UV–VIS spectrophotometer between 400 and 700 nm wavelength. The eluted pigment was concentrated. Further confirmation of bikaverin was performed by nuclear magnetic resonance (NMR) spectrometer. ^1^H NMR spectra were recorded in deutero-chloroform on a Brucker AV 500 MHz NMR spectrometer, Fallanden, Switzerland.

Gibberellic acid (GA_3_) was analyzed by reversed phase HPLC (Themo Separation Products, Fremont, CA, USA) using Lichrosphere100, C_18_, 125 mm × 4 mm column (Merck KGaA, Darmstadt, Germany). The mobile phase used was composed of 20 % acetonitrile in 5 mmol l^−1^ ammonium di-hydrogen phosphate, at pH 2.5; the flow rate used was 0.6 ml min^−1^. Samples were filtered through 0.2 μm membrane filters before injection and introduced into HPLC through a 20 μl loop of Rheodyne manual injector. Gibberellic acid was detected at 205 nm. The quantification was done by external standard method using peak area.

Nitrogen content of the defatted cottonseed meal was determined by flash combustion method using Flash EA, 1112 series, Thermo Finnigan elemental analyzer (Thermo Scientific, Mumbai, India).

The residual glucose in the culture filtrates was estimated by di-nitrosalicyclic acid (DNS) method (Miller 1959).

## Results

### Mutagenesis

The parent strain, *F. fujikuroi* NCIM 1019, grew in filamentous form in shake flask and the broth had reddish violet colour. The UV mutagenesis of this strain generated a wide variety of mutants. An exposure time of 7 s was suitable for 95 % kill and it was used in further mutagenesis. Some of the survivors grew rapidly without any change while others grew slowly and formed small, compact colonies. They exhibited differences in characteristics in terms of size, shape, margin and surface appearance from the parent culture. Along with the morphological changes, a few of them exhibited higher production of the pigment. The small, compact and dry colonies, producing more reddish purple pigment on regeneration agar plates were selected for further study.

### Production of bikaverin by selected mutants of *F. fujikuroi*

When the selected mutants were grown in the basal fermentation medium with 2 g l^−1^ soy bean meal and 50 g l^−1^ sucrose, they exhibited different growth characteristics in the fermentation medium than parent culture. The growth of all these mutants determined as dry cell weight was less as compared to the parent *F. fujikuroi* NCIM 1019. Pigment production started to appear at 24 h and continued to increase in fermentation broths of all the mutants studied.

Table 1 shows that production of both bikaverin and GA_3_ was more in all the selected mutants than that in the parent. All these selected mutants had modified colony morphology as well as microscopic appearance than the parent. A mutant named “Mut-4” produced maximal bikaverin (1715 mg l^−1^) while mutant “Mut-5” produced maximal GA_3_ (132 mg l^−1^). The concentration of bikaverin produced by Mut-4 was considerably higher than all other mutants and the parent. Mut-4 produced 22 times more bikaverin than the parent *F. fujikuroi* NCIM 1019 under identical cultural conditions in shake flask. The growth of all the mutants was almost similar in terms of dry cell mass and thus the specific bikaverin production by Mut-4 was also highest reaching 317 mg bikaverin g^−1^ dry cell weight against just 6.5 mg bikaverin g^−1^ dry cell weight by the parent. Mutant Mut-4 therefore was selected for further optimization of bikaverin production.

**Table 1:** Production of bikaverin by selected mutants of *F. fujikuroi* in 5 day shake flask experiments performed in triplicate

Mutant	DCW g l^−1^	Bikaverin mg l^−1^	Bikaverin mg g^−1^ DCW	GA_3_ mg l^−1^	GA_3_ mg g^−1^ DCW
NCIM 1019	11.8 ± 0.44	77 ± 3.78	6.52	16 ± .66	1.35
Mut-1	6 ± 0.26	525 ± 2.0	87.5	107 ± 5.30	17.8
Mut-2	5.5 ± 0.25	745 ± 4.73	135.0	103 ± 5.30	18.7
Mut-3	6.1 ± 0.30	920 ± 7.93	150.0	55 ± .64	9.0
Mut-4	5.4 ± 0.20	1715 ± 7.81	317.0	41 ± .65	7.6
Mut-5	5.5 ± 0.25	263 ± 7.00	47.8	128 ± 6.03	23.2
Mut-6	5.8 ± 0.30	475 ± 6.55	81.8	132 ± 6.56	22.7
Mut-7	6.2 ± 0.10	912 ± 8.20	147.0	55 ± 2.09	8.8
Mut-8	6 ± 0.26	426 ± 7.21	71.0	45 ± 2.08	7.5
Mut-9	5.7 ± 0.20	520 ± 8.20	91.2	76 ± 3.60	13.3
Mut-10	5.3 ± 0.26	484 ± 7.54	91.3	102 ± 4.16	19.2

**Fig. 2:**
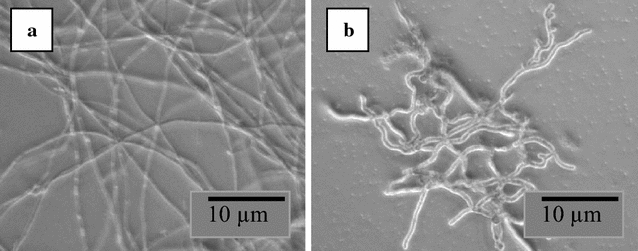
Wild *F. fujikuroi* NCIM 1019 strain and morphological mutant Mut-4 derived from it. Morphological difference as (**a**) long, thin and less branched mycelia of *F. fujikuroi* NCIM 1019 and (**b**) Short, thick and branched mycelia of mutant Mut-4 as seen with ×40 magnification

The microphotographs of the parent strain *F. fujikuroi* NCIM 1019 and Mut-4 (Fig. 2) show that there was a considerable morphological difference in the two strains. Mut-4 grew in short, curled mycelia morphology with extensive branching while the parent hyphae were long and entangled. The liquid cultures of the Mut-4 were much less viscous as compared to the parent because of the short hyphal length.

### Enhancement of bikaverin production in shake flask cultures

Parent strain *F. fujikuroi* NCIM 1019 and mutant Mut-4 were grown in the basal fermentation medium with different concentrations of soy bean meal to study the effect of nitrogen source concentration on their growth, bikaverin and gibberellic acid production. At higher soy bean meal concentrations the apparent viscosity of the fermentation broth considerably increased for parent strain but it was significantly less in Mut-4.

The cell mass of parent and mutant Mut-4 increased with increasing soy bean meal concentration in the medium as seen in Table 2. There was substantial difference between parent and mutant culture in dry cell mass, as well as concentration of bikaverin, GA_3_ and their productivity.

**Table 2:** Effect of defatted soybean meal concentration of bikaverin production by *Fusarium fujikuroi* and mutant Mut-4 in 5 day shake flask experiment

Soybean meal g l^−1^	C:N ratio	*F. fujikuroi* NCIM 1019		Mutant Mut-4	
		DCW g l^−1^	Bikaverin mg g^−1^ DCW	DCW g l^−1^	Bikaverin mg g^−1^ DCW
2	150	12.0 ± 0.4	6.75	5.6 ± 0.17	310.01
4	76	15.5 ± 0.6	3.01	10.2 ± 0.4	87.2
6	51	18.1 ± 0.5	2.08	16.7 ± 0.5	26.95
8	38	19.2 ± 0.7	1.18	18.4 ± 0.55	13.75
10	30	23.0 ± 0.4	0.39	20.2 ± 0.6	7.52

Table 2 shows that at lower soy bean meal concentrations the difference in DCW between the two cultures was much larger. With increase in soy bean meal concentration, the difference became smaller and smaller till 8 g l^−1^ with almost no difference at 10 g l^−1^.

**Fig. 3:**
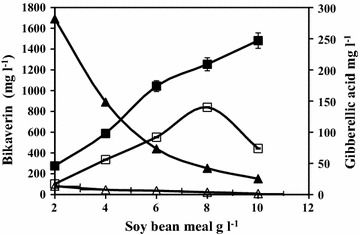
Effect of different concentrations of defatted soy bean meal on bikaverin and gibberellic acid production by *F. fujikuroi* NCIM 1019 and by mutant Mut-4 in 5 day shake flask experiment. *Filled triangle* Bikaverin (mg l^−1^) by mutant Mut-4, *open triangle* Bikaverin (mg l^−1^) by *F. fujikuroi* NCIM 1019, *filled square* GA_3_ (mg l^−1^) by mutant Mut-4, *open square* GA_3_ (mg l^−1^) by *F. fujikuroi* NCIM 1019

Contrary to the expectation, although the cell mass and gibberellic acid concentrations in the shake flasks increased as soy bean meal concentration was increased, bikaverin production declined in both the cultures in the shake flasks (Fig. 3) till 8 g l^−1^ soy meal concentration. Production of gibberellic acid and bikaverin need sufficient oxygen availability. However, bikaverin production was favoured in flasks with lower cell concentration that presumably resulted in ample oxygen availability due to lower broth viscocity. Production of bikaverin and its specific productivity was highest in flasks having lower cell mass in both the cultures. The difference in bikaverin production by Mut-4 in flasks containing different quantities of soy bean meal concentrations employed as nitrogen source was much prominent because of much higher bikaverin production by this selected mutant as compared to the parent fungus.

Figure 3 also shows that unlike bikaverin, GA_3_ concentration in shake flasks increased along with the increase in cell mass caused by use of more nitrogen source. In case of parent culture, GA_3_ concentration and specific production of GA_3_ increased along with the increase in cell mass till 8 g l^−1^ soy bean meal used in the medium but declined at a 10 g l^−1^ soy bean meal concentration. The GA_3_ concentration in case of Mut-4 was relatively higher than the parent and continued to increase even at 10 g l^−1^ soy bean meal concentration.

### Suitable carbon sources for bikaverin production by Mutant Mut-4

**Fig. 4:**
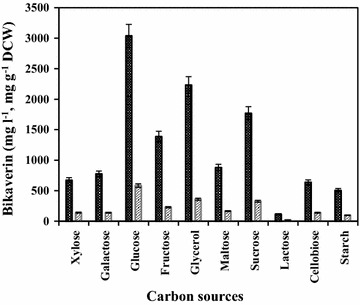
Effect of different carbon sources on production of bikaverin by mutant Mut-4. *Thick bars* indicate bikaverin concentration (mg l^−1^); *light bars* indicate bikaverin specific production (mg g^−1^ DCW). Fermentation was carried out in shake flasks, in triplicate, for 5 days at 28 °C, 200 rpm

To select suitable carbon source for the bikaverin production, Mut-4 was grown in the basal fermentation medium with 2 g l^−1^ soy bean meal and various carbon sources. Varying carbon sources in the culture medium affected bikaverin production by mutant Mut-4 (Fig. 4). The cell mass of the mutant Mut-4 ranged between 4.6 and 6.2 g l^−1^ with different carbon sources used.

Of all the carbon sources studied, accumulation of bikaverin in the fermentation broth was high in media with glucose, fructose, xylose and lactose as could be seen from Additional file 1: Figure S1. Among these four carbon sources, mutant Mut-4 produced substantially higher bikaverin and GA_3_ when glucose was used. When the lactose was used as carbon source, mutant Mut-4 produced comparatively very less bikaverin and GA_3_ although the growth was equal. Higher specific productivity of bikaverin (584 mg bikaverin g^−1^ dry cell weight) and GA_3_ (6.7 mg GA_3_ g^−1^ dry cell weight) in glucose media by mutant Mut-4 was obtained. Consequently, glucose was selected as carbon source for bikaverin production by mutant Mut-4.

### Effect of nitrogen sources on bikaverin production by Mut-4

**Fig. 5:**
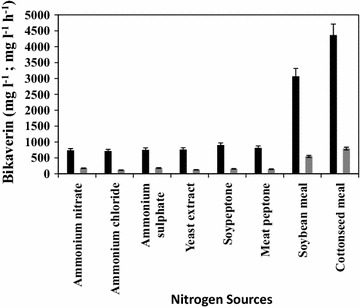
Effect of different nitrogen sources on production of bikaverin by mutant Mut-4. *Solid bars* indicate bikaverin concentration (mg l^−1^); *bars* with *stripes* indicate bikaverin specific production (mg g^−1^ DCW). Fermentation was carried out in shake flasks, in triplicate, for 5 days at 28 °C, 200 rpm

The growth of mutant Mut-4 in terms of dry cell mass was in the range of 4.0–4.2 and 5.5–6.0 g l^−1^ in media containing inorganic and organic nitrogen sources respectively. The amount and the specific productivity of bikaverin by mutant Mut-4 varied within studied nitrogen sources. Figure 5 shows that mutant Mut-4 produced much less bikaverin when the basal medium had inorganic nitrogen sources or soluble, digested protein sources like extracts and peptone.

Figure 5 also shows that amongst studied organic nitrogen sources, mutant Mut-4 produced higher bikaverin in media with defatted plant meals. The specific production of bikaverin in defatted cottonseed meal was 1.4 times more than in defatted soy bean meal. This effect was not due to cell mass, as difference in cell mass of mutant Mut-4 using these defatted plant meals was marginal. Based on the experimental results, a fermentation medium with defatted cottonseed meal as nitrogen source and glucose as the carbon source was formulated and used subsequently.

### Bikaverin production by mutant Mut-4 in cotton seed meal medium

Mutant Mut-4 was cultivated in the modified basal fermentation medium and monitored for its growth, bikaverin and GA_3_ production at every 24 h interval, for 5 days. It grew in the form of short mycelial filaments as observed microscopically and produced abundant red–purple pigment. The pigment was secreted in the medium and was also present intracellualry. The total bikaverin was calculated as bikaverin in the culture filtrate and that extracted from lyophilized dry cell mass.

**Fig. 6:**
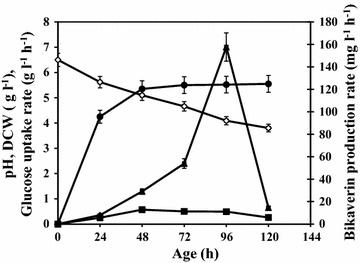
Bikaverin production by mutant Mut-4 in optimized medium *open circle* pH, *filled circle* DCW (g l^−1^), *filled square* Glucose uptake rate (g l^−1^ h^−1^), *filled triangle* Bikaverin production rate (mg l^−1^ h^−1^). Fermentation was carried out in shake flasks, in triplicate, for 5 days at 28 °C, 200 rpm

Figure 6 shows the growth of the mutant Mut-4 and bikaverin production in shake flask culture. Bikaverin as well as gibberellic acid production started after 24 h when the culture entered the stationary phase because of nitrogen exhaustion and continued to increase till end of the fermentation. Most of the increase in the dry cell mass was observed within first 24 h to reach 4.25 g l^−1^ and then it further gradually increased to 5.5 g l^−1^ over next 4 days. There was a gradual decrease in pH to reach 3.5 at the end of 5 days of fermentation. During first 24 h, 6.1 g l^−1^ glucose was consumed from initial 20 g l^−1^ glucose by mutant Mut-4. The glucose uptake rate then rapidly increased and then the glucose was completely exhausted at 48 h of fermentation. At 48 h, 30 g l^−1^ glucose was fed in the form of 50 % glucose solution. Between 48 and 96 h, the average glucose utilization rate in the shake flask culture was 0.5 g l^−1^ h^−1^. At the end of the incubation, glucose was completely consumed by mutant Mut-4. Total bikaverin concentration reached a maximum level of 6335 mg l^−1^ while that of GA_3_ concentration was just 22 mg l^−1^ in 5 days fermentation.

### Analysis of bikaverin and gibberellic acid

The pigment in the cell free broth was extractable with chloroform. It could be precipitated with addition of diethyl ether as described by Bell et al. (2003). Normally bikaverin is associated with second minor compound norbikaverin. TLC plate assay was used to separate bikaverin and norbikaverin. Two bands with R_f_ values of 0.25 and 0.75 were obtained. Based on the results of Bell et al. (2003) these R_f_ values indicated that compounds are norbikaverin and bikaverin, respectively. TLC plates also revealed that the presence of norbikaverin was negligible. The *Rf* value (0.75) and the visible spectrum of this compound indicated that it is bikaverin. Visible spectrum of bikaverin performed using a UV/VIS spectrophotometer was similar to that reported by Balan et al. (1970) and Cornforth et al. (1971) except a small shoulder around 590 nm which may be because of an impurity.

The chemical structure of purified bikaverin was further confirmed by NMR. ^1^H NMR had the following spectra δ 2.86 (s, 3H), δ3.93 (s, 3H), δ3.96 (s, 3H), δ6.37 (s, 1H), δ6.77 (s, 1H) and δ6.92 (s, 1H). This NMR spectrum was identical to that of bikaverin previously reported by Kajer et al. (1971). The compound responsible for the reddish purple colour of the fermentation broth and mycelium of mutant Mut-4 was isolated and thus identified as bikaverin.

Analysis of gibberellic acid (GA_3_) in fermentation broth was confirmed using an on-line spectral scanning detector during HPLC analysis. The 3D spectral scanning was performed between 200 and 350 nm. It showed a peak purity index of 999 for the standard GA_3_ peak and had maximal absorbance at 205 nm. The GA_3_ peak in the chromatogram of the fermentation broth had identical spectral pattern and peak purity index to that of the standard GA_3_.

## Discussion

Polyketide pigments are produced by a variety of fungi and have been studied for their biological activity as well as a characteristic in fungal taxonomy. Because of their toxic nature, these pigments were considered to be antibiotics by earlier investigators. Polyketides were found to play protective role in case of environmental stress in certain fungi (Medentsev et al. 2005). Like most of the *Fusarium* species, *F. fujikuroi* has a complex metabolic capability and produces diverse secondary metabolites like gibberellins, carotenoids and polyketides of very diverse chemical nature. Bikaverin is a polyketide of biotechnological interest and like gibberellins, it is also secreted by *F. fujikuroi* into the culture medium. In our previous study (Lale et al. 2006), we had reported mutants of *F. fujikuroi* that had short, highly branched hyphae, and curly tips with thick, swollen ends which grew with lower viscosity of fermentation broth and resulted in increased production of gibberellic acid. Although gibberellins and bikaverin are extracellular, it is not easy to select colonies after mutagenesis for enhanced gibberellins because of absence of suitable plate assays. In the present investigation however, selection of mutants for enhanced bikaverin biosynthesis was relatively simple due to production of intense water soluble red pigment by the colonies that diffused in regeneration agar plates. From the several mutants studied, a mutant “Mut-4” was selected and studied based on the higher bikaverin production and high specific productivity. Mut-4 is a morphological mutant that also grows in thick, short and highly branched mycelium and results in lower viscosity of the fermentation broth. Several other mutants also had similar morphology and lower viscosity but only Mut-4 showed distinctly higher bikaverin production. Although the altered morphology does not have a direct correlation with the enhanced production of bikaverin, the increased production of bikaverin by Mut-4 may have been supported further because of lower viscosity of fermentation broth that allowed increased oxygen transfer as compared to parent *F. fujikuroi* 1019.

The source of nitrogen and its concentration in the fermentation medium play a very significant role in growth of the cultures and the production of secondary metabolites. Excessive quantity of nitrogen source prolongs the growth phase and the cultures can become oxygen limited which can prove to be detrimental to the secondary metabolite production. It was reported that bikaverin and gibberellin biosynthesis are repressed by high nitrogen concentrations (Linnemannstöns et al. 2002) while higher aeration and lower nitrogen content (C:N ratio 150:1) enhanced bikaverin production in the fermentation broth by *F. fujikuroi* (Giordano et al. 1999; Escamilla-Silva et al. 2001). On this basis, in the present investigation, the effect of different concentrations of soy bean meal on growth as well as bikaverin and gibberellic acid production was investigated. Defatted soy bean meal is a common nitrogen source used for production of secondary metabolites including antibiotics and was used initially for the bikaverin production in the present investigation. When Mut-4 was studied for bikaverin production in media with different quantities of soy bean meal as nitrogen source, it was observed that a medium low in soy bean meal that resulted in lesser cell mass was more suitable for bikaverin production. Thus, a basal fermentation medium with 2 g l^−1^ soy bean meal and 50 g l^−1^ sucrose with C:N ratio (150:1) was formulated and used for bikaverin production subsequently. Normally the higher cell mass generated through use of higher concentrations of nutrients is expected to result in higher quantity of any fermentation product. But in the present investigation, Mut-4 was found to produce maximal bikaverin in a fermentation medium with very low nitrogen source concentration. Keeping all other culture conditions identical, the shake flask culture with lower cell mass must have experienced higher dissolved oxygen and presumably this resulted in higher bikaverin production. The requirement of high dissolved oxygen in submerged fermentation of polyketide compounds natamycin (Liang et al. 2008), lovastatin (Rodriguez-Porcel et al. 2006) and erythromycin (Zou et al. 2008) to achieve high productivity has been reported and supports our findings in the case of bikaverin production. This difference indicated the possibility of a specific aeration-sensitive bikaverin production regulation, controlling its biosynthesis.

Using different culture conditions, earlier investigators have reported that bikaverin production was strongly enhanced by the presence of sucrose in comparison with other carbon sources in *F. fujikuroi* cultures (Rodriguez-Ortiz et al. 2010) while in *Fusarium oxysporum* f sp. *vasinfectum*, maltose increased bikaverin production under nitrogen starvation (Bell et al. 2003). In the present investigation, the maximum production of bikaverin (3039 mg l^−1^) and higher specific production (584 mg bikaverin g^−1^ DCW) was obtained in medium containing glucose as the carbon source. This difference could be due to difference in experimental conditions and regulatory mechanisms leading to bikaverin production between these strains.

During investigation on culture conditions of mutant Mut-4 it was observed that the production of bikaverin as well as gibberellic acid was governed by quantity of nitrogen source in the media. The concentration and type of the nitrogen has to be chosen properly keeping in mind the oxygen transfer rate of any culture vessel may it be shake flask or agitated fermenter. A variety of nitrogen sources were investigated for production of bikaverin by mutant Mut-4 and it was found that complex organic nitrogen sources substantially enhanced bikaverin production. When soy peptone, a papaic digest of soy bean meal, was used as a nitrogen source on equal nitrogen basis, it resulted in the production of much less bikaverin as compared to the defatted soy bean meal. Among the used organic nitrogen sources, defatted cotton seed meal supported maximum bikaverin production. This result emphasizes the need of insoluble and complex nitrogen sources for fungal fermentations for production of secondary metabolites. Thus it was not merely an effect of total nitrogen content of the fermentation medium. Although it is a common observation that secondary metabolite production is enhanced when complex organic nitrogen sources are used in the medium, the exact role of such nitrogen sources is not clear. It is possible that the stimulation in bikaverin production and higher productivity by mutant Mut-4 in the medium containing natural agricultural residue such as defatted cottonseed meal could be due to the presence of precursors of the bikaverin pathway. Although other investigators have used ammonium salts and glycine as favourable nitrogen source for bikaverin production, it was reported that the growth and bikaverin production was comparatively less in media containing inorganic nitrogen sources than organic nitrogen sources (Bell et al. 2003).

Biosynthesis of bikaverin and gibberellins in *F. fujikuroi* is observed only after the depletion of nitrogen source in the culture medium (Candau et al. 1992). In the present investigation, when mutant Mut-4 was cultivated in optimized fermentation media containing glucose and defatted cottonseed meal, bikaverin as well as gibberellic acid production was first observed at 24 h when culture entered the stationary phase. Wiemann et al. (2009) reported that biosynthesis of bikaverin was time dependant and reached maximum at 72 h. In present study also, production of bikaverin peaked between 72 and 96 h. There was substantial difference observed between GA_3_ and bikaverin concentration and their specific production by mutant Mut-4. Bikaverin production rate increased from 29 to 157.7 mg l^−1^ h^−1^ between 48 and 96 h although glucose utilization rate remained nearly constant during this period. This also indicated that there was no direct correlation between glucose consumption rate and bikaverin production rate. Mut-4 produced substantially high bikaverin (6335 mg l^−1^) in 5 day shake flask culture compared to the earlier reports. This production was two times higher than that reported in shake flask cultures and three times more than reported in solid substrate fermentation.

In conclusion, bikaverin production by *F. fujikuroi* mutant Mut-4 was influenced by the medium composition, especially by the type and concentration of nitrogen source whereas suitability of the carbon source for bikaverin production seems to be strain dependent. It also depends largely on dissolved oxygen tension. The present investigation is successful in achieving enhanced production of bikaverin by a mutant of *F. fujikuroi* by optimizing media constituents.

## Additional file

10.1186/s13568-016-0205-0 Production of bikaverin in shake flasks by* Fusarium fujikuroi* Mut‐4 using different carbon sources
