# Supplementary material for: Production of bikaverin by a Fusarium fujikuroi mutant in submerged cultures
Source: AMB Express. 2016 May 3;6:34. doi: 10.1186/s13568-016-0205-0 (PMC4854849; doi:10.1186/s13568-016-0205-0)
Supplement: Supplementary file 1 — 10.1186/s13568-016-0205-0 Production of bikaverin in shake flasks by Fusarium fujikuroi Mut‐4 using different carbon sources [file 13568_2016_205_MOESM1_ESM.pdf]

AMB Express

Production of bikaverin by a *Fusarium fujikuroi* mutant in submerged cultures

G. J. Lale and R.V. Gadre*

Biochemical and Biological Engineering Group,
Chemical Engineering and Process Development Division
CSIR-National Chemical Laboratory
Pune 411008
India

*Correspondence to
Dr. R. V. Gadre, Chemical Engineering and Process Development Division
National Chemical Laboratory, Pune, 411008, India
E-mail: rv.gadre@ncl.res.in
Telephone: +91 20 25902348
Fax: +91 20 25902612

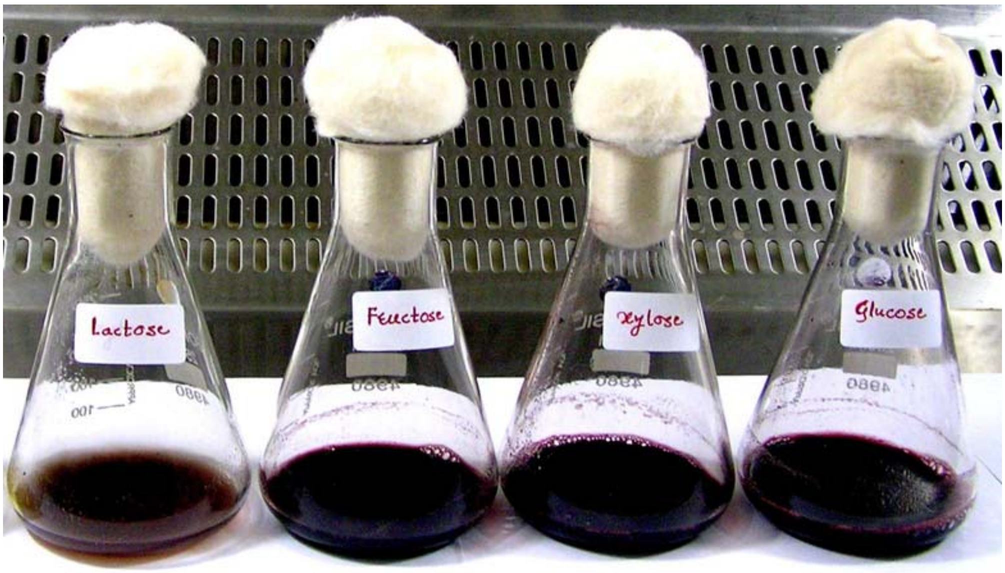

Figure S1. Production of bikaverin in shake flasks by *Fusarium fujikuroi* Mut-4 using different carbon sources

Fusarium fujikuroi mutant Mut-4 was grown in 50 ml fermentation medium with 2 g l^{-1} soybean meal as nitrogen source and lactose, glucose, fructose and xylose as carbon sources at 50 g l^{-1} concentration. The flasks were incubated at 28 °C for 120 h, at 200 rpm. The bikaverin production by mutant Mut-4 was very intense in media with glucose, fructose and xylose, while lactose resulted in lower bikaverin production.
